# (2*E*)-3-(2-Chloro-8-methyl­quinolin-3-yl)-1-(2-methyl-4-phenyl­quinolin-3-yl)prop-2-en-1-one

**DOI:** 10.1107/S1600536813020229

**Published:** 2013-07-27

**Authors:** R. Prasath, S. Sarveswari, Seik Weng Ng, Edward R. T. Tiekink

**Affiliations:** a Department of Chemistry, BITS, Pilani – K. K. Birla Goa Campus, Goa 403 726, India; b Centre for Organic and Medicinal Chemistry, VIT University, Vellore 632 014, India; c Department of Chemistry, University of Malaya, 50603 Kuala Lumpur, Malaysia; d Chemistry Department, Faculty of Science, King Abdulaziz University, PO Box 80203 Jeddah, Saudi Arabia

## Abstract

In the title compound, C_29_H_21_ClN_2_O, there is a twist in the bridging prop-2-en-1-one group [C=C—C=O torsion angle = 22.7 (2)°]. The quinolinyl residues form a dihedral angle of 86.92 (4)°, indicating an almost perpendicular relationship. In the crystal, supra­molecular layers in the *bc* plane are stabilized by C—H⋯π and π–π inter­actions [centroid–centroid distance = 3.4947 (7) Å].

## Related literature

For background details and the biological applications of quinolinyl chalcones, see: Joshi *et al.* (2011 ▶); Prasath & Bhavana (2012 ▶); Prasath *et al.* (2013*a* ▶). For a related structure, see: Prasath *et al.* (2013*b* ▶).
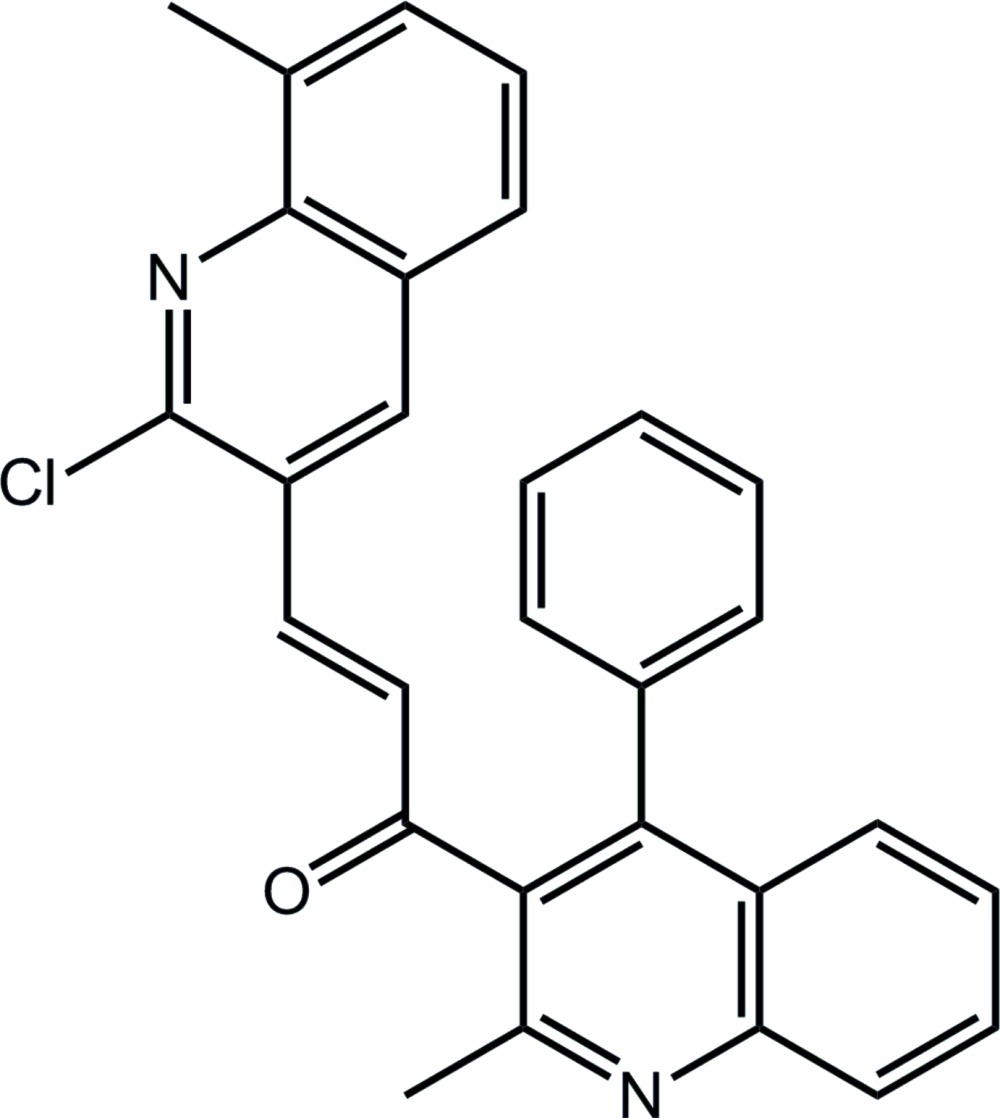


## Experimental

### 

#### Crystal data

C_29_H_21_ClN_2_O
*M* *_r_* = 448.93
Monoclinic, 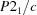

*a* = 10.9837 (2) Å
*b* = 21.0604 (3) Å
*c* = 9.3927 (1) Å
β = 90.009 (1)°
*V* = 2172.73 (6) Å^3^
*Z* = 4
Cu *K*α radiation
μ = 1.75 mm^−1^
*T* = 100 K
0.35 × 0.15 × 0.10 mm

#### Data collection

Agilent SuperNova Dual diffractometer with an Atlas detector
Absorption correction: multi-scan (*CrysAlis PRO*; Agilent, 2013 ▶) *T*_min_ = 0.852, *T*_max_ = 1.000
8885 measured reflections
4444 independent reflections
3956 reflections with *I* > 2σ(*I*)
*R*_int_ = 0.020

#### Refinement

*R*[*F* ^2^ > 2σ(*F* ^2^)] = 0.034
*wR*(*F* ^2^) = 0.093
*S* = 1.03
4444 reflections
300 parameters
H-atom parameters constrained
Δρ_max_ = 0.26 e Å^−3^
Δρ_min_ = −0.35 e Å^−3^

### 

Data collection: *CrysAlis PRO* (Agilent, 2013 ▶); cell refinement: *CrysAlis PRO*; data reduction: *CrysAlis PRO*; program(s) used to solve structure: *SHELXS97* (Sheldrick, 2008 ▶); program(s) used to refine structure: *SHELXL97* (Sheldrick, 2008 ▶); molecular graphics: *ORTEP-3 for Windows* (Farrugia, 2012 ▶) and *DIAMOND* (Brandenburg, 2006 ▶); software used to prepare material for publication: *publCIF* (Westrip, 2010 ▶).

## Supplementary Material

Crystal structure: contains datablock(s) general, I. DOI: 10.1107/S1600536813020229/hg5335sup1.cif

Structure factors: contains datablock(s) I. DOI: 10.1107/S1600536813020229/hg5335Isup2.hkl

Supplementary material file. DOI: 10.1107/S1600536813020229/hg5335Isup3.cml

Additional supplementary materials:  crystallographic information; 3D view; checkCIF report

## Figures and Tables

**Table 1:** Hydrogen-bond geometry (Å, °) *Cg*1 and *Cg*2 are the centroids of the C1–C6 and N1,C1,C6-C9 rings, respectively.

*D*—H⋯*A*	*D*—H	H⋯*A*	*D*⋯*A*	*D*—H⋯*A*
C13—H13⋯*Cg*1^i^	0.95	2.90	3.5847 (15)	130
C16—H16⋯*Cg*2^ii^	0.95	2.74	3.6060 (14)	152

Symmetry codes: (i) 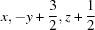; (ii) 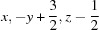.

**Fig. 1.**
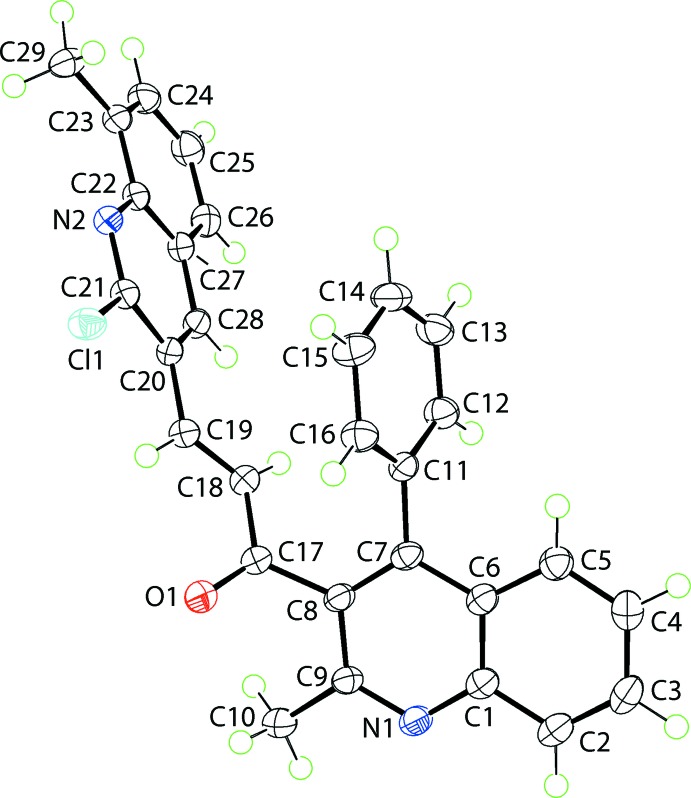
The molecular structure of (I) showing the atom-labelling scheme and displacement ellipsoids at the 70% probability level.

**Fig. 2.**
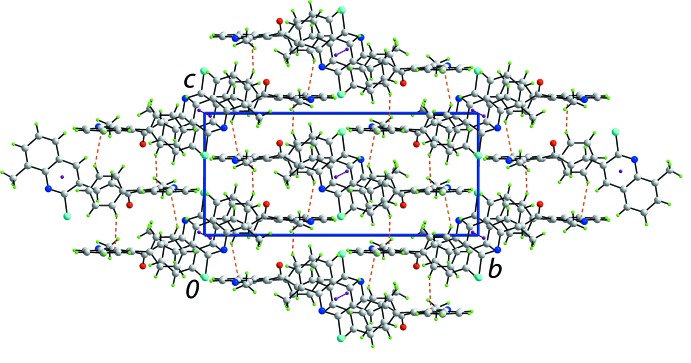
View of the supramolecular layer formed in the bc plane by π—π and C—H···π interactions shown as purple and orange dashed lines, respectively.

**Fig. 3.**
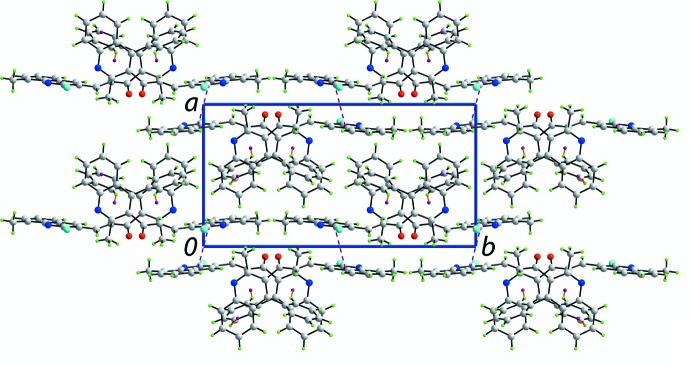
View in projection down the c axis of the unit-cell contents of (I). The π—π and C—H···π interactions are shown as purple and orange dashed lines, respectively.

## supplementary crystallographic information

### Comment

Nitrogen-containing heterocyclic analogues are found to be key intermediates in organic synthesis and exhibit a multitude of biological properties (Prasath & Bhavana, 2012). This has prompted research into the design and synthesis of a variety of nitrogen-containing chalcone derivatives, and their evaluation for anti-bacterial, anti-fungal, anti-malarial and anti-cancer potential (Prasath *et al.*, 2013*a*; Joshi *et al.*, 2011). It was in this connection that the title compound, (I), was investigated.

The molecular structure of (I), Fig. 1, comprises two quinolinyl residues connected by the ends of a prop-2-en-1-one bridge. The dihedral angle between the quinolinyl residues is 86.92 (4)°, indicating an almost perpendicular relationship. The phenyl ring is inclined with respect to the quinolinyl residue to which it is attached, forming a dihedral angle of 72.70 (5)°. The conformation about the ethylene bond [C18═C19 = 1.3363 (18) Å] is *E*. A twist in the bridging prop-2-en-1-one group is manifested in the O1—C17—C18—C19 torsion angle of 22.7 (2)°. An similar open conformation was reported recently for a related structure, namely (2*E*)-3-(2-chloro-8-methylquinolin-3-yl)-1-(2,4-dimethylquinolin-3-yl)prop-2-en-1-one (Prasath *et al.*, 2013*b*).

In the crystal packing, π—π interactions between centrosymmetrically related N2-pyridyl rings [centroid···centroid distance = 3.4947 (7) Å and symmetry operation: -*x*, 1 - *y*, 1 - *z*] combine with phenyl-C—H···π interactions, Table 1, to stabilize supramolecular layers in the *bc* plane, Fig. 2. Layers inter-digitate along the *a* axis with no specific interactions between them, Fig. 3.

### Experimental

A mixture of 3-acetyl-2-methyl-4-phenylquinoline (260 mg, 0.001 *M*), 2-chloro-8-methylquinoline-3-carbaldehyde (200 mg, 0.001 *M*) and KOH (0.2 g) in methanol (20 ml) was stirred for 12 h at room temperature. The resulting mixture was neutralized with dilute acetic acid. The deposited solid was filtered, dried and purified by column chromatography using a 1:1 mixture of ethyl acetate and hexane. Re-crystallization was by slow evaporation of an acetone solution of (I); 81% yield, *M*.pt: 381–383 K.

### Refinement

Carbon-bound H-atoms were placed in calculated positions [C—H = 0.95–0.98 Å, *U*_iso_(H) = 1.2–1.5*U*_eq_(C)] and were included in the refinement in the riding model approximation.

### Crystal data

**Table:**

C_29_H_21_ClN_2_O	*F*(000) = 936
*M**_r_* = 448.93	*D*_x_ = 1.372 Mg m^−^^3^
Monoclinic, *P*2_1_/*c*	Cu *K*α radiation, λ = 1.54184 Å
Hall symbol: -P 2ybc	Cell parameters from 5025 reflections
*a* = 10.9837 (2) Å	θ = 4.0–76.5°
*b* = 21.0604 (3) Å	µ = 1.75 mm^−^^1^
*c* = 9.3927 (1) Å	*T* = 100 K
β = 90.009 (1)°	Prism, pale-yellow
*V* = 2172.73 (6) Å^3^	0.35 × 0.15 × 0.10 mm
*Z* = 4	

### Data collection

**Table:**

Agilent SuperNova Dual diffractometer with an Atlas detector	4444 independent reflections
Radiation source: SuperNova (Cu) X-ray Source	3956 reflections with *I* > 2σ(*I*)
Mirror monochromator	*R*_int_ = 0.020
Detector resolution: 10.4041 pixels mm^-1^	θ_max_ = 76.6°, θ_min_ = 4.0°
ω scan	*h* = −13→13
Absorption correction: multi-scan (*CrysAlis PRO*; Agilent, 2013)	*k* = −26→17
*T*_min_ = 0.852, *T*_max_ = 1.000	*l* = −11→9
8885 measured reflections	

### Refinement

**Table:**

Refinement on *F*^2^	Primary atom site location: structure-invariant direct methods
Least-squares matrix: full	Secondary atom site location: difference Fourier map
*R*[*F*^2^ > 2σ(*F*^2^)] = 0.034	Hydrogen site location: inferred from neighbouring sites
*wR*(*F*^2^) = 0.093	H-atom parameters constrained
*S* = 1.03	*w* = 1/[σ^2^(*F*_o_^2^) + (0.0517*P*)^2^ + 0.5734*P*] where *P* = (*F*_o_^2^ + 2*F*_c_^2^)/3
4444 reflections	(Δ/σ)_max_ < 0.001
300 parameters	Δρ_max_ = 0.26 e Å^−^^3^
0 restraints	Δρ_min_ = −0.35 e Å^−^^3^

### Special details

**Table:**

Geometry. All s.u.'s (except the s.u. in the dihedral angle between two l.s. planes) are estimated using the full covariance matrix. The cell s.u.'s are taken into account individually in the estimation of s.u.'s in distances, angles and torsion angles; correlations between s.u.'s in cell parameters are only used when they are defined by crystal symmetry. An approximate (isotropic) treatment of cell s.u.'s is used for estimating s.u.'s involving l.s. planes.
Refinement. Refinement of *F*^2^ against ALL reflections. The weighted *R*-factor *wR* and goodness of fit *S* are based on *F*^2^, conventional *R*-factors *R* are based on *F*, with *F* set to zero for negative *F*^2^. The threshold expression of *F*^2^ > 2σ(*F*^2^) is used only for calculating *R*-factors(gt) *etc*. and is not relevant to the choice of reflections for refinement. *R*-factors based on *F*^2^ are statistically about twice as large as those based on *F*, and *R*- factors based on ALL data will be even larger.

### Fractional atomic coordinates and isotropic or equivalent isotropic displacement parameters (Å^2^)

**Table:**

	*x*	*y*	*z*	*U*_iso_*/*U*_eq_	
Cl1	0.12153 (3)	0.498061 (14)	0.15385 (3)	0.02104 (10)	
O1	0.07264 (9)	0.72584 (5)	0.24609 (12)	0.0279 (2)	
N1	0.25624 (10)	0.88805 (5)	0.38792 (11)	0.0194 (2)	
N2	0.15106 (9)	0.42899 (5)	0.37948 (11)	0.0170 (2)	
C1	0.37937 (12)	0.88017 (6)	0.37540 (13)	0.0178 (3)	
C2	0.45334 (13)	0.93537 (6)	0.37127 (14)	0.0220 (3)	
H2	0.4167	0.9762	0.3758	0.026*	
C3	0.57705 (13)	0.93017 (7)	0.36081 (14)	0.0236 (3)	
H3	0.6257	0.9675	0.3584	0.028*	
C4	0.63343 (12)	0.87007 (7)	0.35355 (14)	0.0224 (3)	
H4	0.7194	0.8671	0.3451	0.027*	
C5	0.56435 (12)	0.81582 (6)	0.35870 (13)	0.0201 (3)	
H5	0.6030	0.7755	0.3550	0.024*	
C6	0.43568 (12)	0.81954 (6)	0.36946 (13)	0.0173 (2)	
C7	0.35840 (11)	0.76506 (6)	0.36811 (13)	0.0164 (2)	
C8	0.23401 (12)	0.77416 (6)	0.36914 (13)	0.0173 (2)	
C9	0.18560 (12)	0.83726 (6)	0.38431 (13)	0.0181 (3)	
C10	0.05163 (13)	0.84859 (6)	0.40364 (16)	0.0253 (3)	
H10A	0.0387	0.8909	0.4444	0.038*	
H10B	0.0181	0.8164	0.4680	0.038*	
H10C	0.0107	0.8459	0.3112	0.038*	
C11	0.41321 (11)	0.70034 (6)	0.36303 (13)	0.0167 (2)	
C12	0.47280 (12)	0.67678 (6)	0.48298 (14)	0.0208 (3)	
H12	0.4828	0.7030	0.5644	0.025*	
C13	0.51759 (13)	0.61511 (7)	0.48404 (15)	0.0234 (3)	
H13	0.5567	0.5990	0.5667	0.028*	
C14	0.50532 (13)	0.57701 (6)	0.36472 (15)	0.0240 (3)	
H14	0.5351	0.5347	0.3658	0.029*	
C15	0.44938 (14)	0.60088 (6)	0.24349 (15)	0.0246 (3)	
H15	0.4428	0.5751	0.1608	0.030*	
C16	0.40293 (12)	0.66235 (6)	0.24261 (14)	0.0203 (3)	
H16	0.3642	0.6784	0.1596	0.024*	
C17	0.14745 (12)	0.72006 (6)	0.34199 (14)	0.0197 (3)	
C18	0.16008 (12)	0.66060 (6)	0.42449 (14)	0.0194 (3)	
H18	0.1953	0.6611	0.5169	0.023*	
C19	0.12123 (12)	0.60618 (6)	0.36714 (14)	0.0193 (3)	
H19	0.0789	0.6091	0.2792	0.023*	
C20	0.13765 (11)	0.54270 (6)	0.42670 (14)	0.0173 (2)	
C21	0.13793 (11)	0.48749 (6)	0.33840 (13)	0.0168 (2)	
C22	0.16629 (11)	0.41804 (6)	0.52263 (13)	0.0166 (2)	
C23	0.17783 (11)	0.35408 (6)	0.56986 (14)	0.0189 (3)	
C24	0.19252 (12)	0.34365 (6)	0.71322 (15)	0.0226 (3)	
H24	0.2006	0.3012	0.7464	0.027*	
C25	0.19608 (13)	0.39384 (7)	0.81299 (14)	0.0237 (3)	
H25	0.2065	0.3847	0.9113	0.028*	
C26	0.18456 (12)	0.45557 (6)	0.76904 (14)	0.0207 (3)	
H26	0.1867	0.4892	0.8364	0.025*	
C27	0.16941 (11)	0.46883 (6)	0.62186 (14)	0.0177 (3)	
C28	0.15474 (11)	0.53112 (6)	0.56974 (14)	0.0182 (3)	
H28	0.1567	0.5658	0.6344	0.022*	
C29	0.17284 (13)	0.30021 (6)	0.46518 (15)	0.0241 (3)	
H29A	0.1849	0.2599	0.5155	0.036*	
H29B	0.2371	0.3056	0.3938	0.036*	
H29C	0.0933	0.2999	0.4179	0.036*	

### Atomic displacement parameters (Å^2^)

**Table:**

	*U*^11^	*U*^22^	*U*^33^	*U*^12^	*U*^13^	*U*^23^
Cl1	0.02611 (17)	0.01835 (16)	0.01868 (16)	0.00250 (11)	−0.00140 (12)	0.00135 (11)
O1	0.0274 (5)	0.0181 (5)	0.0383 (6)	−0.0019 (4)	−0.0141 (4)	0.0028 (4)
N1	0.0228 (6)	0.0147 (5)	0.0207 (5)	−0.0007 (4)	−0.0013 (4)	0.0008 (4)
N2	0.0157 (5)	0.0145 (5)	0.0209 (5)	−0.0003 (4)	−0.0007 (4)	−0.0001 (4)
C1	0.0224 (6)	0.0160 (6)	0.0150 (6)	−0.0014 (5)	−0.0009 (4)	0.0003 (4)
C2	0.0278 (7)	0.0157 (6)	0.0224 (6)	−0.0033 (5)	−0.0001 (5)	0.0005 (5)
C3	0.0273 (7)	0.0196 (6)	0.0238 (6)	−0.0085 (5)	−0.0010 (5)	0.0006 (5)
C4	0.0196 (6)	0.0264 (7)	0.0212 (6)	−0.0041 (5)	−0.0008 (5)	0.0004 (5)
C5	0.0221 (6)	0.0190 (6)	0.0190 (6)	−0.0008 (5)	−0.0014 (5)	−0.0008 (5)
C6	0.0212 (6)	0.0159 (6)	0.0147 (6)	−0.0016 (5)	−0.0014 (4)	−0.0001 (4)
C7	0.0217 (6)	0.0137 (6)	0.0138 (5)	−0.0005 (5)	−0.0015 (4)	0.0009 (4)
C8	0.0207 (6)	0.0131 (6)	0.0181 (6)	−0.0013 (5)	−0.0020 (5)	0.0015 (4)
C9	0.0203 (6)	0.0141 (6)	0.0198 (6)	−0.0002 (5)	−0.0023 (5)	0.0024 (5)
C10	0.0209 (7)	0.0170 (6)	0.0381 (8)	0.0024 (5)	−0.0010 (6)	0.0011 (6)
C11	0.0161 (6)	0.0141 (6)	0.0200 (6)	−0.0011 (5)	0.0009 (4)	0.0011 (5)
C12	0.0239 (6)	0.0192 (6)	0.0192 (6)	0.0013 (5)	−0.0025 (5)	−0.0004 (5)
C13	0.0239 (7)	0.0219 (7)	0.0245 (7)	0.0034 (5)	−0.0024 (5)	0.0049 (5)
C14	0.0260 (7)	0.0164 (6)	0.0296 (7)	0.0056 (5)	0.0025 (5)	0.0017 (5)
C15	0.0322 (7)	0.0179 (6)	0.0238 (7)	0.0032 (5)	0.0015 (5)	−0.0033 (5)
C16	0.0242 (6)	0.0185 (6)	0.0184 (6)	0.0022 (5)	−0.0006 (5)	0.0008 (5)
C17	0.0187 (6)	0.0138 (6)	0.0266 (7)	0.0001 (5)	−0.0022 (5)	−0.0004 (5)
C18	0.0178 (6)	0.0160 (6)	0.0245 (6)	−0.0013 (5)	−0.0017 (5)	0.0018 (5)
C19	0.0176 (6)	0.0150 (6)	0.0253 (6)	0.0003 (5)	−0.0012 (5)	0.0013 (5)
C20	0.0134 (5)	0.0138 (6)	0.0246 (6)	−0.0011 (4)	−0.0008 (4)	−0.0005 (5)
C21	0.0152 (6)	0.0163 (6)	0.0189 (6)	−0.0001 (5)	−0.0006 (4)	0.0007 (5)
C22	0.0130 (5)	0.0158 (6)	0.0209 (6)	−0.0002 (4)	−0.0003 (4)	0.0008 (5)
C23	0.0159 (6)	0.0153 (6)	0.0255 (6)	−0.0002 (5)	−0.0002 (5)	0.0016 (5)
C24	0.0206 (6)	0.0193 (6)	0.0279 (7)	0.0003 (5)	−0.0012 (5)	0.0061 (5)
C25	0.0229 (7)	0.0276 (7)	0.0205 (6)	−0.0009 (5)	−0.0024 (5)	0.0047 (5)
C26	0.0194 (6)	0.0221 (6)	0.0206 (6)	−0.0013 (5)	−0.0010 (5)	−0.0015 (5)
C27	0.0137 (6)	0.0168 (6)	0.0225 (6)	−0.0011 (5)	−0.0005 (4)	−0.0002 (5)
C28	0.0158 (6)	0.0154 (6)	0.0234 (6)	−0.0010 (5)	−0.0004 (5)	−0.0032 (5)
C29	0.0288 (7)	0.0140 (6)	0.0293 (7)	0.0009 (5)	−0.0017 (5)	0.0007 (5)

### Geometric parameters (Å, º)

**Table:**

Cl1—C21	1.7568 (13)	C13—C14	1.385 (2)
O1—C17	1.2252 (16)	C13—H13	0.9500
N1—C9	1.3219 (16)	C14—C15	1.3879 (19)
N1—C1	1.3677 (17)	C14—H14	0.9500
N2—C21	1.2991 (16)	C15—C16	1.3916 (18)
N2—C22	1.3744 (16)	C15—H15	0.9500
C1—C2	1.4188 (18)	C16—H16	0.9500
C1—C6	1.4198 (18)	C17—C18	1.4792 (17)
C2—C3	1.367 (2)	C18—C19	1.3363 (18)
C2—H2	0.9500	C18—H18	0.9500
C3—C4	1.411 (2)	C19—C20	1.4605 (17)
C3—H3	0.9500	C19—H19	0.9500
C4—C5	1.3723 (18)	C20—C28	1.3783 (18)
C4—H4	0.9500	C20—C21	1.4282 (17)
C5—C6	1.4191 (18)	C22—C27	1.4193 (17)
C5—H5	0.9500	C22—C23	1.4238 (17)
C6—C7	1.4273 (17)	C23—C24	1.3738 (19)
C7—C8	1.3797 (18)	C23—C29	1.5023 (18)
C7—C11	1.4909 (17)	C24—C25	1.413 (2)
C8—C9	1.4384 (17)	C24—H24	0.9500
C8—C17	1.5057 (17)	C25—C26	1.3699 (19)
C9—C10	1.5018 (18)	C25—H25	0.9500
C10—H10A	0.9800	C26—C27	1.4201 (18)
C10—H10B	0.9800	C26—H26	0.9500
C10—H10C	0.9800	C27—C28	1.4095 (18)
C11—C16	1.3900 (18)	C28—H28	0.9500
C11—C12	1.3942 (18)	C29—H29A	0.9800
C12—C13	1.3887 (18)	C29—H29B	0.9800
C12—H12	0.9500	C29—H29C	0.9800
			
C9—N1—C1	118.69 (11)	C14—C15—H15	119.9
C21—N2—C22	117.60 (11)	C16—C15—H15	119.9
N1—C1—C2	117.98 (12)	C11—C16—C15	120.05 (12)
N1—C1—C6	122.91 (12)	C11—C16—H16	120.0
C2—C1—C6	119.10 (12)	C15—C16—H16	120.0
C3—C2—C1	120.37 (13)	O1—C17—C18	122.14 (12)
C3—C2—H2	119.8	O1—C17—C8	118.21 (12)
C1—C2—H2	119.8	C18—C17—C8	119.52 (11)
C2—C3—C4	120.78 (12)	C19—C18—C17	119.01 (12)
C2—C3—H3	119.6	C19—C18—H18	120.5
C4—C3—H3	119.6	C17—C18—H18	120.5
C5—C4—C3	120.17 (13)	C18—C19—C20	126.25 (12)
C5—C4—H4	119.9	C18—C19—H19	116.9
C3—C4—H4	119.9	C20—C19—H19	116.9
C4—C5—C6	120.48 (12)	C28—C20—C21	114.95 (11)
C4—C5—H5	119.8	C28—C20—C19	123.51 (12)
C6—C5—H5	119.8	C21—C20—C19	121.54 (12)
C5—C6—C1	119.10 (12)	N2—C21—C20	126.86 (12)
C5—C6—C7	123.18 (12)	N2—C21—Cl1	115.12 (10)
C1—C6—C7	117.66 (11)	C20—C21—Cl1	118.01 (10)
C8—C7—C6	118.49 (11)	N2—C22—C27	121.26 (11)
C8—C7—C11	121.81 (11)	N2—C22—C23	118.32 (11)
C6—C7—C11	119.68 (11)	C27—C22—C23	120.42 (12)
C7—C8—C9	119.68 (11)	C24—C23—C22	117.85 (12)
C7—C8—C17	121.26 (11)	C24—C23—C29	121.67 (12)
C9—C8—C17	118.84 (11)	C22—C23—C29	120.48 (12)
N1—C9—C8	122.21 (12)	C23—C24—C25	122.25 (12)
N1—C9—C10	116.32 (11)	C23—C24—H24	118.9
C8—C9—C10	121.41 (11)	C25—C24—H24	118.9
C9—C10—H10A	109.5	C26—C25—C24	120.50 (12)
C9—C10—H10B	109.5	C26—C25—H25	119.8
H10A—C10—H10B	109.5	C24—C25—H25	119.8
C9—C10—H10C	109.5	C25—C26—C27	119.40 (12)
H10A—C10—H10C	109.5	C25—C26—H26	120.3
H10B—C10—H10C	109.5	C27—C26—H26	120.3
C16—C11—C12	119.40 (12)	C28—C27—C22	118.08 (12)
C16—C11—C7	121.30 (11)	C28—C27—C26	122.31 (12)
C12—C11—C7	119.28 (11)	C22—C27—C26	119.59 (12)
C13—C12—C11	120.32 (12)	C20—C28—C27	121.25 (12)
C13—C12—H12	119.8	C20—C28—H28	119.4
C11—C12—H12	119.8	C27—C28—H28	119.4
C14—C13—C12	120.10 (12)	C23—C29—H29A	109.5
C14—C13—H13	119.9	C23—C29—H29B	109.5
C12—C13—H13	119.9	H29A—C29—H29B	109.5
C13—C14—C15	119.81 (12)	C23—C29—H29C	109.5
C13—C14—H14	120.1	H29A—C29—H29C	109.5
C15—C14—H14	120.1	H29B—C29—H29C	109.5
C14—C15—C16	120.27 (13)		
			
C9—N1—C1—C2	176.45 (12)	C7—C11—C16—C15	−176.59 (13)
C9—N1—C1—C6	−4.90 (19)	C14—C15—C16—C11	0.5 (2)
N1—C1—C2—C3	179.11 (12)	C7—C8—C17—O1	−124.57 (14)
C6—C1—C2—C3	0.41 (19)	C9—C8—C17—O1	50.12 (18)
C1—C2—C3—C4	0.2 (2)	C7—C8—C17—C18	51.55 (18)
C2—C3—C4—C5	−0.8 (2)	C9—C8—C17—C18	−133.76 (13)
C3—C4—C5—C6	0.84 (19)	O1—C17—C18—C19	22.7 (2)
C4—C5—C6—C1	−0.26 (19)	C8—C17—C18—C19	−153.30 (13)
C4—C5—C6—C7	176.77 (12)	C17—C18—C19—C20	173.33 (12)
N1—C1—C6—C5	−179.00 (11)	C18—C19—C20—C28	24.6 (2)
C2—C1—C6—C5	−0.37 (18)	C18—C19—C20—C21	−154.88 (13)
N1—C1—C6—C7	3.81 (18)	C22—N2—C21—C20	0.07 (19)
C2—C1—C6—C7	−177.56 (11)	C22—N2—C21—Cl1	178.95 (9)
C5—C6—C7—C8	−175.52 (11)	C28—C20—C21—N2	0.82 (19)
C1—C6—C7—C8	1.55 (17)	C19—C20—C21—N2	−179.62 (12)
C5—C6—C7—C11	3.39 (18)	C28—C20—C21—Cl1	−178.04 (9)
C1—C6—C7—C11	−179.54 (11)	C19—C20—C21—Cl1	1.52 (16)
C6—C7—C8—C9	−5.52 (18)	C21—N2—C22—C27	−0.93 (17)
C11—C7—C8—C9	175.59 (11)	C21—N2—C22—C23	178.49 (11)
C6—C7—C8—C17	169.12 (11)	N2—C22—C23—C24	−179.74 (11)
C11—C7—C8—C17	−9.77 (18)	C27—C22—C23—C24	−0.32 (18)
C1—N1—C9—C8	0.64 (19)	N2—C22—C23—C29	−0.26 (18)
C1—N1—C9—C10	177.94 (11)	C27—C22—C23—C29	179.17 (11)
C7—C8—C9—N1	4.64 (19)	C22—C23—C24—C25	0.16 (19)
C17—C8—C9—N1	−170.14 (12)	C29—C23—C24—C25	−179.32 (12)
C7—C8—C9—C10	−172.53 (12)	C23—C24—C25—C26	0.1 (2)
C17—C8—C9—C10	12.70 (18)	C24—C25—C26—C27	−0.2 (2)
C8—C7—C11—C16	68.30 (17)	N2—C22—C27—C28	0.85 (18)
C6—C7—C11—C16	−110.58 (14)	C23—C22—C27—C28	−178.55 (11)
C8—C7—C11—C12	−109.78 (14)	N2—C22—C27—C26	179.61 (11)
C6—C7—C11—C12	71.35 (16)	C23—C22—C27—C26	0.21 (18)
C16—C11—C12—C13	−2.3 (2)	C25—C26—C27—C28	178.78 (12)
C7—C11—C12—C13	175.77 (12)	C25—C26—C27—C22	0.07 (19)
C11—C12—C13—C14	1.2 (2)	C21—C20—C28—C27	−0.85 (17)
C12—C13—C14—C15	0.7 (2)	C19—C20—C28—C27	179.60 (12)
C13—C14—C15—C16	−1.6 (2)	C22—C27—C28—C20	0.09 (18)
C12—C11—C16—C15	1.5 (2)	C26—C27—C28—C20	−178.63 (12)

### Hydrogen-bond geometry (Å, º)

Cg1 and Cg2 are the centroids of the C1–C6 and N1,C1,C6-C9 rings, respectively.

**Table:**

*D*—H···*A*	*D*—H	H···*A*	*D*···*A*	*D*—H···*A*
C13—H13···*Cg*1^i^	0.95	2.90	3.5847 (15)	130
C16—H16···*Cg*2^ii^	0.95	2.74	3.6060 (14)	152

Symmetry codes: (i) *x*, −*y*+3/2, *z*+1/2; (ii) *x*, −*y*+3/2, *z*−1/2.
